# Development of ST-246® for Treatment of Poxvirus Infections

**DOI:** 10.3390/v2112409

**Published:** 2010-11-03

**Authors:** Robert Jordan, Janet M. Leeds, Shanthakumar Tyavanagimatt, Dennis E. Hruby

**Affiliations:** SIGA Technologies, 4575 SW Research Way, Corvallis, OR 97333, USA; E-Mails: jleeds@siga.com (J.M.L); shanthak@siga.com (S.T.); dhruby@siga.com (D.E.H.)

**Keywords:** Smallpox, ST-246, Tecovirimat, orthopoxvirus, p37, egress inhibitor, antiviral drug

## Abstract

ST-246 (Tecovirimat) is a small synthetic antiviral compound being developed to treat pathogenic orthopoxvirus infections of humans. The compound was discovered as part of a high throughput screen designed to identify inhibitors of vaccinia virus-induced cytopathic effects. The antiviral activity is specific for orthopoxviruses and the compound does not inhibit the replication of other RNA- and DNA-containing viruses or inhibit cell proliferation at concentrations of compound that are antiviral. ST-246 targets vaccinia virus p37, a viral protein required for envelopment and secretion of extracellular forms of virus. The compound is orally bioavailable and protects multiple animal species from lethal orthopoxvirus challenge. Preclinical safety pharmacology studies in mice and non-human primates indicate that ST-246 is readily absorbed by the oral route and well tolerated with the no observable adverse effect level (NOAEL) in mice measured at 2000 mg/kg and the no observable effect level (NOEL) in non-human primates measured at 300 mg/kg. Drug substance and drug product processes have been developed and commercial scale batches have been produced using Good Manufacturing Processes (GMP). Human phase I clinical trials have shown that ST-246 is safe and well tolerated in healthy human volunteers. Based on the results of the clinical evaluation, once a day dosing should provide plasma drug exposure in the range predicted to be antiviral based on data from efficacy studies in animal models of orthopoxvirus disease. These data support the use of ST-246 as a therapeutic to treat pathogenic orthopoxvirus infections of humans.

## 1. Human Orthopoxvirus Infections

Human orthopoxviruses cause a spectrum of diseases, ranging from severe disseminated lesional disease characteristic of the most common type of variola virus infection (variola major) to localized lesional infection caused by vaccinia virus. Of the several species of orthopoxvirus known to infect humans, variola virus, the etiological agent of smallpox, is by far the most virulent. Four major types of clinical disease are associated with variola virus infection and are defined by the morphology of the virus-specific lesion and severity of disease symptoms. Ordinary smallpox is characterized by raised pustular skin lesions that can be confluent or discrete. Variola sine eruptione is characterized by fever without rash and requires serological analysis to confirm diagnosis. Flat type smallpox is characterized by confluent flat pustules, and hemorrhagic type smallpox is characterized by widespread hemorrhages in the skin and mucous membranes. Both flat type and hemorrhagic type smallpox are usually fatal with 97% mortality in diagnosed cases [1]. A less severe form of variola virus infection (variola minor) has been observed during outbreaks characterized by less severe lesional disease and lower mortality. The molecular etiology of reduced disease severity of variola minor is not well understood.

Three other species of orthopoxviruses (monkeypox, vaccinia, cowpox viruses) have been found to infect humans and cause disease. Monkeypox virus causes a generalized infection in humans resembling a milder version of smallpox. Monkeypox is a zoonotic disease that is endemic in some areas of the Democratic Republic of the Congo (DRC) [2,3]. The virus is poorly transmissible from person to person and outbreaks are self-limiting, resulting in small numbers of people contracting disease [2]. Vaccinia virus, the primary component of the currently licensed smallpox vaccine, is a laboratory strain with no known natural reservoir. Vaccinia virus causes localized infection in humans when administered percutaneously and generates protective immunity against variola virus [1,4]. Vaccinia-like viruses have been isolated from patients in Brazil. Patients infected with cowpox or vaccinia-like viruses present with localized lesions of the hands and arms similar to vaccinia virus infection [5,6]. Human orthopoxviruses are believed to be maintained in the population through rodent reservoirs and subclinical (and clinical) zoonotic infections occur with high frequency through contact with infected animals, or through an intermediate species such as cattle or domestic pets [2,7]. In Ghana, orthopoxvirus antibodies were detected in 53% of the people living in proximity to forest dwelling rodent populations, which also tested positive for orthopoxvirus exposure based on antibody titers and PCR analysis of selected tissue samples [8]. Disease severity in all cases is influenced by host immune status. Individuals suffering from certain skin disorders or who are immunocompromised suffer more severe infection [9–12].

Other species of orthopoxviruses that are genetically related to variola virus such as camelpox and ectromelia viruses have not been found to infect humans. The genetic basis for susceptibility of poxviruses is not well understood, but is thought to be related to acquisition and adaptive evolution of host response modifier genes [13,14]. These genes are often found to be virulence factors that down regulate the host immune response and thereby facilitate systemic virus spread. Phylogenetic analysis of poxvirus genomes has identified a number of gene families undergoing positive selection, many of which are candidate host response modifier genes [15]. Recent outbreaks of fatal cowpox virus infections in non-human primates coupled with the observation that host-range genes and virulence factors are undergoing positive selection suggest that orthopoxviruses are evolving, leading to increased zoonotic transmission and variants with altered virulence [16]. While smallpox is no longer a disease found in humans, the possibility exists that new variants of circulating orthopoxviruses may emerge to cause more frequent disease in humans. Thus, there is a need for new therapeutics to treat pathogenic orthopoxvirus infections.

## 2. Discovery of ST-246 (Tecovirimat)

In early 2002, prompted by a biodefense initiative launched by the National Institute of Allergy and Infectious disease (NIAID), a high throughput screening (HTS) assay was developed to quantify vaccinia and cowpox virus-induced cytopathic effects (CPE) in vero cell cultures. This CPE-based assay was used to evaluate 356,240 chemical compounds from a diverse collection of chemical scaffolds for their ability to inhibit orthopoxvirus-induced CPE. Compounds that inhibited virus-induced CPE by greater than 50% relative to untreated virus controls at a compound concentration of 5 μM were evaluated further. At total of 759 hits were discovered (0.2% hit rate) and grouped into nine distinct chemical series based upon the structure of their parent scaffolds. Several chemical series were optimized further based on nascent Structure Activity Relationships (SAR) of related analogs [17].

Compound potency was evaluated from dose response curves generated by measuring virus-induced CPE in the presence of a range of compound concentrations. The effective concentration of each compound that protected cell monolayers by 50% (EC_50_) from virus-induced CPE was calculated from the dose response curve.

**Figure 1.**
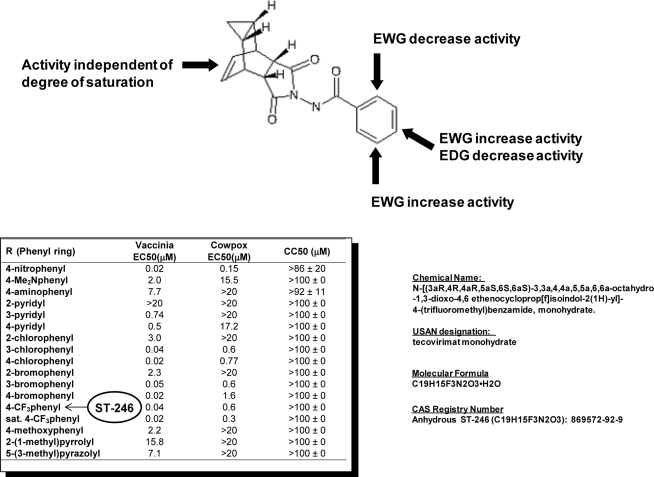
Structure activity relationships and chemical information for ST-246. A summary of antiviral activity of chemical analogs of ST-246 (adapted from Bailey *et al.* [18]). EWG—Electron withdrawing groups; EDG—Electron donating groups. R refers to modifications of the phenyl ring.

Examination of hits from the HTS assay revealed a series of tricyclononene carboxamides (Figure 1) with EC_50_ values that ranged from 20 nM to the upper limit of measurement (>20 μM). Nascent structure activity relationships (SAR) indicated that electron withdrawing substitution on the carboxamide aryl or heteroaryl enhanced potency of the molecules in the CPE assay. To validate these nascent structure activity relationships, a series of analogs were prepared, and tested against both vaccinia and cowpox viruses in cell-based CPE assays [18].

The SAR demonstrated that electron withdrawing substitution on the carboxamide carbonyl R-group provided the most potent inhibitor compounds (Figure 1). This was exemplified by the 4-nitrophenyl substituted carboxamide, which was 100-fold more potent than the electron-donating 4-dimethylaminophenyl analog against both vaccinia and cowpox viruses. While the aza-π-deficient 3- and 4-pyridyl displayed potency against vaccinia, the 2-pyridyl analog displayed a dramatic loss of potency. In all cases, heterocyclic substitution provided modest to weak potency against vaccinia, and no potency against cowpox. For the chloro- and bromo-substituted phenyls, a similar pattern was observed for both vaccinia and cowpox where 3- and 4-substitution was more potent than 2-substitution. Reduction of the olefin had little effect on potency [18].

*In vitro* metabolic stability assays of selected analogs from this chemical series were conducted to assess the potential for *in vivo* stability. The 4-trifluoromethyl phenol derivative (designated ST-246) was selected for further characterization from a group of analogs based on relative metabolic stability.

## 3. Preclinical Pharmacology

### 3.1. Selectivity

ST-246 exhibited potent antiviral activity against a broad spectrum of orthopoxviruses in CPE assays while showing little activity against unrelated RNA and DNA containing viruses [17]. The EC_50_ values for inhibition of viral replication ranged from 0.01 μM for vaccinia virus to 0.07 μM for ectromelia virus to greater than 40 μM for unrelated viruses. Notably, cowpox appears to be less susceptible to ST-246 when compared on the same cell lines (5 to 50-fold) [19]. The mechanism of reduced susceptibility to ST-246 is unknown but may reflect a different mode of virus spread that is less dependent upon formation of extracellular virus. ST-246 was active against a CDV-resistant (CDVr) cowpox virus (EC_50_ = 0.05 μM), suggesting that the mechanism by which ST-246 inhibits virus replication is distinct from that of CDV. Furthermore, ST-246 inhibited clinical isolates from both of the major clades of monkeypox and variola viruses in cell culture [20]. ST-246 inhibited orthopoxvirus replication in a variety of cell types including human embryonic lung fibroblasts, primary human keratinocytes, and organotypic endothelial raft cultures [19].

### 3.2. Cellular Toxicity

The cytotoxicity of ST-246 was measured in selected cell lines from mouse, rabbit, monkey, and humans. Cell viability was determined by measuring the reduction of alamar blue (resazurin) by fluorescence spectroscopy or by direct cell counting. The CC_50_ values were found to be >50 μM in all cell lines tested including human embryonic lung fibroblasts and primary human keratinocytes [19]. In addition, the growth rate of HEK-293, L929, MRC5, and SIRC cells, measured over a 72 hour time period in the presence and absence of 50 uM ST-246 for three days was reduced by 30–40% relative to untreated controls. Growth of vero and BSC40 cells was not affected by ST-246 treatment [21]

### 3.3. Mechanism of Action

**Figure 2.**
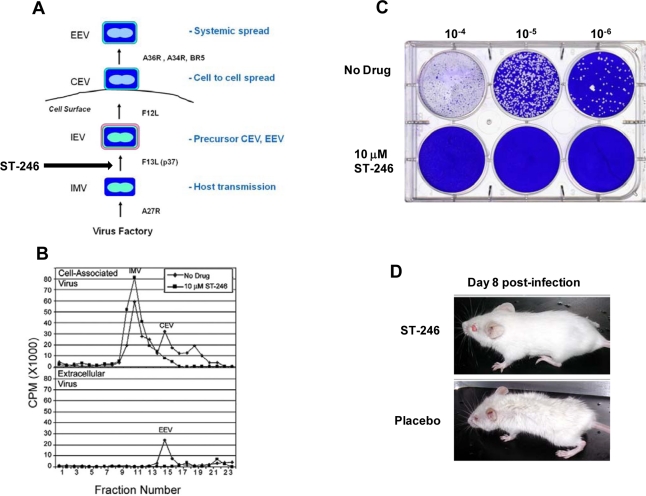
ST-246 inhibits production of extracellular virus and systemic virus spread *in vitro* and *in vivo*. **(A)** Diagram showing the four infectious forms of vaccinia virus and viral genes required for each step in the morphogenesis process. Vaccinia virus genes involved in each step in the pathway are shown (adapted from Smith *et al.* [23]). **(B)** ST-246 inhibits extracellular virus (CEV and EEV) formation. BSC-40 cell monolayers were infected with vaccinia virus at 5 pfu/cell in the presence and absence of 10 μM ST-246. The cell monolayers were radiolabeled with ^35^-S methionine and vaccinia virus particles, either cell-associated (upper graph) or released into the culture medium (lower graph) were fractionated by equilibrium centrifugation on cesium chloride gradients. The radiolabeled material in each fraction was quantified by liquid scintillation. The assignment of each type of virus particle was based upon their reported density (adapted from Chen *et al.*) [48] **(C)** ST-246 inhibits plaque formation. BSC-40 cell monolayers (1 × 10^6^ cells/well) were infected with 10-fold serial dilutions of vaccinia virus in the presence and absence of 5 μM ST-246. At 3 days post-infection, the cultures were fixed in 5% glutaraldehyde and stained with crystal violet to visualize plaques. **(D)** ST-246 protects mice from systemic disease. Mice were inoculated with a lethal dose of vaccinia virus (WR) via the intranasal route. ST-246 was administered at 100 mg/kg as a liquid suspension by oral gavage once per day for 14 days. Shown are mice treated with ST-246 or placebo at day 8 post-infection.

Orthopoxviruses are large double-stranded DNA viruses that replicate exclusively in the cytoplasm of infected cells. There are four types of infectious virus particles produced during productive infection; intracellular mature virus (IMV), intracellular enveloped virus (IEV), cell associated enveloped virus (CEV), and extracellular enveloped virus (EEV) (Figure 2A). The intracellular and extracellular forms of the virus are thought to play unique roles in orthopoxvirus pathogenesis [22,23].

IMV particles assemble from crescent-shaped membranes in virus factory areas of the cytoplasm. Particle formation requires a series of temporally regulated proteolytic cleavage events of viral core proteins that result in condensation of the viral core [24]. The core particles are enveloped by intracellular membranes to form IMV particles. Once formed, these particles remain inside the cell and are released upon cell lysis. IMV particles are stable in the environment and are thought to play a role in host transmission [23].

Approximately ten percent (10%) of the total infectious particles produced during infection are wrapped in virus modified membranes derived from post-trans Golgi or endosomal membrane systems to form IEV particles [25]. Once formed, IEV particles travel to the cell surface in a microtubule-dependent fashion where the outer membrane of the IEV containing vesicle fuses with the plasma membrane to release CEV that remain associated with the cell surface. Approximately one percent (1%) of CEV particles are released into the extracellular space as EEV particles by a process involving motile actin tail formation [26,27].

The extracellular virus particles (EEV and CEV) are responsible for efficient cell-to-cell spread and long range dissemination of virus in the host [25,28,29]. Virus variants containing defects in genes required for production of extracellular virus particles produce small plaques *in vitro* and are attenuated for virus spread *in vivo* [30–32]. Passive immunization with antibodies directed against IMV particles are less protective than antibodies directed against whole virus, suggesting that neutralizing antibodies to EEV antigens play a significant role in disease prevention [33]. Finally, inhibitors of extracellular virus formation or more specifically, production of EEV particles, protect animals from lethal orthopoxvirus infection [17,34,35]. These observations suggest that extracellular virus particles play an important role in virus spread and orthopoxvirus disease progression.

At least seven virus-specific gene products are required for synthesis of extracellular virus particles. These gene products participate in the wrapping of IMV particles (B5R and F13L), transport of particles to the cell surface (F12L), actin tail formation (A33R, A34R, and A36R), and release of particles from the cell (A33R, A34R, and B5R). EEV particles have a higher specific infectivity compared to IMV particles and are more resistant to complement mediated neutralization [36,37]. Complement resistance is the result of incorporation of host complement control proteins, CD46, CD55, and CD59, into the outer membrane of the virus [36]. These properties of extracellular virus particles facilitate long-range spread of the virus in the host.

The target of ST-246 has been identified through mapping genetic resistance to the vaccinia virus F13L gene. Vaccinia virus recombinants containing deletions in F13L are not sensitive to ST-246 suggesting that F13L is the sole target of ST-246 activity. These variants produce a small plaque phenotype, fail to produce extracellular virus, and are attenuated for replication in mouse models of vaccinia virus infection. F13L gene encodes a highly conserved 37 KDa peripheral membrane protein (p37) required for the production of extracellular forms of virus [25,38]. Indeed, ST-246 inhibits extracellular virus production and plaque formation consistent with known activities associated with p37 (Figure 2B and 2C).

ST-246 prevents formation of extracellular virus by inhibiting the formation of a putative wrapping complex derived from virus-modified late endosomal (LE) membranes. The wrapping complex catalyzes the envelopment of intracellular mature virus particles to produce egress-competent forms of the virus [23,39–41]. The formation of the wrapping complex requires the activities of p37 and other viral proteins that interact with membrane proteins associated with the LE [23,40]. The LE is enriched for Rab9 protein that mediates recycling of cation-dependent mannose-6-phosphate receptor (CD-MPR) [42] and cation-independent mannose-6-phosphate receptor (CI-MPR) from the LE to the trans Golgi network (TGN) [43]. Rab9-dependent recycling is mediated through interactions with the tail interacting protein of 47 kD (TIP47), a Rab9-specific effector [44,45]. TIP47 binds to a proline-rich motif found within the C-terminus of CI-MPR and a diaromatic (Tyr-Trp) motif found within the cytosolic tail of CD-MPR [43]. Likewise, TIP47 has also been shown to interact with the HIV Env protein through a similar diaromatic (Tyr-Trp) motif [46,47]. RNAi-mediated depletion of Rab9 inhibited replication of human immunodeficiency virus type 1, filoviruses, and measles virus consistent with Rab9-containing vesicles playing a role in virus assembly [48].

The putative wrapping complex is thought to be formed through interactions of p37 with components of LE-derived transport vesicles [49]. Immunoprecipitation studies have demonstrated that p37 associates with Rab9 and TIP47 in membrane fractions from infected cells and that these interactions are essential for plaque formation [49]. Moreover, ST-246 blocks the interaction of p37 with Rab9 and TIP47, suggesting that the compound inhibits formation of the wrapping complex. These results suggest that p37 and other viral proteins involved in extracellular virus formation, may substitute for cellular cargo to induce Rab9-TIP47-dependent vesicle formation. These vesicles contain p37 and B5R protein and resemble the virus modified-membrane precursors or post-TGN vesicles required for envelopment described previously [40,41]. These results support the idea of a common pathway used by viruses that assemble at the LE whereby virus envelope proteins act as cargo to concentrate and recruit host effector and Rab proteins to stimulate envelopment of virus particles. The exact mechanism by which ST-246 inhibits IMV wrapping has not been fully elucidated.

## 4. Metabolism and Pharmacokinetics

### 4.1. In vitro Absorption, Distribution, Metabolism, and Excretion (ADME)

ST-246 is poorly soluble in aqueous solution but shows good Caco-2 membrane permeability categorizing ST-246 as a biopharmaceutical classification system 2 drug. The evaluation of ST-246 ADME properties showed it has moderate to high plasma protein binding, is relatively stable and not metabolized to any significant degree by any cytochrome P450 isozymes. There was no significant cytochrome P450 (CYP) induction at 10 μM ST-246 (3,700 ng/mL) but at 100 μM induction was observed for the following CYP enzymes: 2B6, 2C9, 3A4, and 2C19. Inhibition of nine CYP enzymes that were tested (CYP1A2, CYP2B6, CYP2C8, CYP2C9, CYP2C19, CYP2D6, CYP2E1, and CYP3A4) was less than 50% at a concentration of 300 μM. Thus, the potential for drug-drug interactions via induction or inhibition of human cytochrome p450 enzymes is low. The *in vitro* patch clamp assay for potential hERG channel inhibition showed interaction with the potassium ion channel was quite low. The highest ST-246 concentration, 30 μM, showed only 7% inhibition.

### 4.2. Metabolism

While the evaluation of metabolic stability in isolated microsomes showed that small amounts of ST-246 were cleaved, liberating 4-trifluoromethylbenzoic acid from the parent compound in rats, mice and dogs, these metabolites were not seen in either the monkey or human microsomes. No other metabolites were identified from the *in vitro* studies. An *in vivo* mass balance study in mice showed that radioactivity associated with ^14^C-ST-246 was nearly completely eliminated within 96 hours after oral administration. At 24 hours post dose, the radioactivity was broadly distributed to all organs, including the brain, with the highest concentration outside of the intestinal tract observed in the gallbladder. By 96 hours after oral administration, approximately 72% of the radioactivity had been eliminated through the feces and 24% was eliminated in the urine. Whereas all of the radiolabel in the feces co-eluted on HPLC with intact ST-246 none of the radiolabel in the urine did. The urine contained multiple metabolites; however, the only ones that could be identified, in addition to trifluorobenzoic acid, were glucuronidated ST-246 metabolites.

### 4.3. Pharmacokinetics

**Figure 3.**
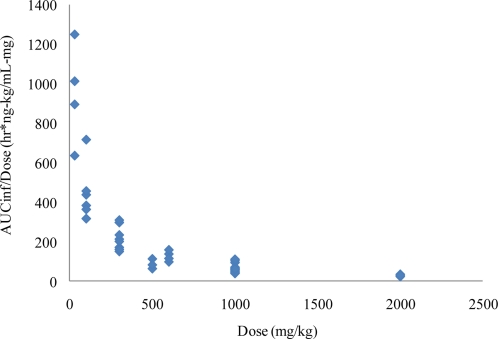
Dose normalized exposure of ST-246 in BALB/c mice from Day 1 of multiple studies. Dose normalized area-under-the curve values are plotted *versus* dose for mouse toxicology studies. The results suggest that absorption limits increased exposure as the dose is increased. AUC_inf_ = Area under concentration time curve for plasma drug levels after oral dosing from time 0 extrapolated to infinite time.

The nonclinical pharmacokinetic profile of ST-246 was evaluated in several *in vivo* studies in BALB/c mice, Spraque-Dawley rats, New Zealand White rabbits and Cynomolgus monkeys. Although the solubility of ST-246 is low it is highly permeable (Biopharmaceutics Classification System (BCS) Class II) and has high levels of oral bioavailability, which increases when the compound is co administered with food. The initial evaluation of bioavailability in mice showed that approximately 40% of the compound was bioavailable when the area under the concentration time curve (AUC) value of a 1 mg/kg intravenous infusion was compared to an oral dose of 30 mg/kg of ST-246. Higher doses had lower apparent bioavailability. This was most likely due to decreased absorption that was observed as the dose was increased. In rats, the bioavailability was 90% and 33%, respectively, for males and females after oral administration of 30 mg/kg ST-246. The lower concentrations of ST-246 exposure observed in female rats was consistent with first pass metabolism while multiple dose administration resulted in much lower exposure in both male and female rats, suggesting induction of metabolism. Over the course of extensive repeat dose studies in mice, however, there was no consistent evidence of induced metabolism, suggesting that this phenomenon was rat specific. The predominant cause of nonlinearity in the pharmacokinetics of ST-246 observed in mice was the apparent decreased absorption with increasing dose. The decreased absorption was observed in both the observed maximum plasma concentrations as well as the exposure (as determined by AUC values). Thus, as the doses were increased, exposures also increased but not dose proportionally (Figure 3).

Due to this apparent saturation of absorbance of ST-246 in mice the apparent clearance and volume of distribution increased with increasing doses after oral administration. The compound was dosed once a day with the terminal elimination half-life in mice of approximately four hours. In the 28 day repeat dose study, mice showed a modest amount of accumulation over the course of the study for the higher two of the three doses, approximately 25% increase in AUC values at the 1000 mg/kg dose and approximately 50% at the 2000 mg/kg dose. However, in the 90 day study the AUC values were lower on Day 90 than Day 1 by 25% to 50%.

As was observed in mice, the bioavailability in monkeys increased when the animals were fed, doubling from approximately 25% when animals had been fasted to roughly 50% when the animals were administered ST-246 in the fed state. Dose proportional exposure was observed at lower doses but, as mentioned above, it appeared that absorption may have limited exposure increases at higher doses. In the 28 day study, the exposure at the end of the study tended to be lower than on Day 1 of the study, with values that ranged from 25% of the Day 1 exposure to equivalent exposure. In contrast, in the three month monkey study, the exposure at the last time point was nearly double that observed on the first day of the study. Thus, in multiple dose studies in both mice and monkeys there was no strong evidence of significant accumulation of ST-246 nor induction of metabolism. It may be that the differences observed in the different studies were more related to the different absorption properties of dose formulation suspensions used on a given day than of any metabolic differences between the animals throughout the course of the safety studies.

The steady state volume of distribution in monkeys was calculated after a 1 mg/kg IV dose administered during the initial bioavailability study. The values for both male and female monkeys were greater than the total body water volume in monkeys (693 mL/kg [50]), indicating a broad distribution of the compound, consistent with the radiolabel distribution and mass balance study in mice. The clearance for both male and female monkeys was close to 1100 mL/hr/kg. Although in some studies there were statistical trends indicating gender differences in the pharmacokinetics, as with the level of accumulation during multi-dose studies, these differences were not consistently observed.

## 5. Safety Evaluation

### 5.1. Genotoxicity

ST-246 was not genotoxic in either bacterial or mammalian cell genotoxicity assays. In addition ST-246 did not cause chromosomal damage or bone marrow toxicity in the mouse micronucleus test. Because of the completely negative results in the evaluation of genotoxicity tests carcinogenicity tests have not been done nor are they anticipated or warranted.

### 5.2. Safety Pharmacology

Safety pharmacology studies for ST-246 were conducted. Plethysmography was used to measure tidal volume in mice after single oral dose administration of ST-246 of up to 2000 mg/kg. There were no abnormal values associated with the administration of ST-246 and the no observable effect level (NOEL) was determined to be the highest administered dose, or 2000 mg/kg. An initial study in mice used the Functional Observational Battery (FOB) to evaluate the potential for CNS toxicity. As with the pulmonary safety pharmacology, the top dose of 2000 mg/kg in mice did not elicit any behavioral or other functional changes such as core body temperature. However, studies in dogs found an association between ST-246 administration and behavioral and electroencephalogram (EEG) evidence of seizures. The maximum tolerated dose in dogs, based on this observed toxicity, was 30 mg/kg. A follow up study in primates found no evidence of altered electroencephalograms or lowered seizure thresholds in NHP after administration of 12 daily doses of 300 mg/kg of ST-246 indicating that dogs are uniquely sensitive to ST-246. In addition, analysis of brain and CSF levels of ST-246 were found to be much higher in dogs than NHP, suggesting that the toxicity was specific to dogs. Cardiovascular safety evaluation in NHP showed no prolongation of QTc at the highest dose administered in repeat dose studies, 300 mg/kg.

### 5.3. General Toxicology

SIGA has conducted safety evaluations in multiple species, mice, rats, rabbits, dogs, and NHP. The two species used for repeat dose toxicity studies were mice and NHP, as these species are also used in efficacy evaluations so that a thorough knowledge of safety in these species helped with the design of efficacy and pharmacology studies. The maximum tolerated dose in mice was 2000 mg/kg for both single dose as well as the 28 Day study. In the single dose study there was a slight loss of body weight but recovery by the second day. Although both liver and spleen weights were slightly increased at the end of the 28 Day study in mice for the 2000 mg/kg dose of ST-246 there were no corresponding histological correlates and no alterations in clinical chemistry or liver enzymes. The no observable adverse effect level (NOAEL) was 2000 mg/kg. The three month study evaluated a top dose of 1000 mg/kg of ST-246 in mice and the same slight increase in liver weight with no accompanying histopathology was observed, meaning the NOAEL was 1000 mg/kg for the three month study. The highest single dose administered orally to NHP was 2000 mg/kg, which resulted in decreased activity and ataxia in both male and female NHP. This was also observed after single dose administration of 1000 mg/kg. At 300 mg/kg ataxia was observed four hours post dose in one female NHP. Because of these observations the highest dose used in multiple dose safety studies in NHP was 300 mg/kg. This was used for both the 28 Day and 3 Month Safety studies and the NOEL was the top dose of 300 mg/kg in both of these studies. ST-246 is a well-tolerated compound and seems to elicit very little toxicity at doses that are much higher than those required for the antiviral activity.

### 5.4. Reproductive Toxicology

Definitive Segment I, II, and III reproductive studies were conducted in both mice and rabbits. There was no evidence in either mice or rabbits of decreased fertility or fetal resorptions, fetal abnormalities or toxicity. The highest dose used in the rabbit Segment II study, 100 mg/kg, resulted in some maternal toxicity. Administration of ^14^C-ST-246 to pregnant mice resulted in only very low levels of radiolabel in the placenta and to nursing dams very low levels (<1%) in the milk.

## 6. Chemistry Manufacturing and Control

ST-246® is a tetracyclic acylhydrazide compound being developed for treatment of pathogenic orthopoxvirus infections. ST-246 is a small synthetic molecule with a molecular weight of 376.33 g/mol. The calculated logP (log of the octanol-water partition coefficient) for ST-246 is about 2.94 and it has a melting point of 196 °C. ST-246 is very soluble in organic solvents such as methanol, ethanol and acetonitrile and is sparingly soluble in water and simulated gastro-intestinal fluids. Based on its low solubility in aqueous fluid and good partition coefficient, ST-246 can be classified as a BCS class II drug per US-FDA definition. ST-246 is non-hygroscopic and is a chemically stable molecule. It does not undergo degradation under normal room temperature storage conditions. Stability studies per International Conference on Harmonisation (ICH) guidelines are in progress and data available to date demonstrate that ST-246 is stable for up to two years at controlled room temperature.

ST-246 is a white to off-white crystalline powder and exhibits polymorphism. Polymorphic screening using various solvents suggests that ST-246 can exist in three major physical forms: anhydrous, hemihydrate and monohydrate. Physico-chemical properties of all the forms were found to be similar. Manufacturing processes have been developed to produce all the forms of ST-246 consistently.

ST-246 drug substance is manufactured via a four-stage convergent process consisting of three bond-forming chemical reactions together with four solid isolations, one of which is highly purifying. Process has been demonstrated from small scale (gram level) to full commercial scale (>1000 kg). The process is rugged and produces high quality (>99.0%) pure material. SIGA has produced several small batches, three NDA one-tenth commercial (NDA (New Drug Application) registration batches) scale and is in the process of full commercial scale process validation.

Comprehensive Quality by Design (QbD) study per ICH Q8 was completed to identify an appropriate Design Space for each step of the manufacturing process. The ICH Q9 Quality Risk Assessment has also been conducted to identify Focus Areas in the each step of the manufacturing process. In general, typically Focus Areas (FAs) included the reaction, work-up, solvent displacement(s), crystallization and drying. This work also supported the critical process parameter study. The early stages of the study included a Quality Risk Assessment, per ICH Q9, to identify FAs that aided in designing the multivariate experiments. This approach satisfied criteria outlined in ICH Q8 and Q9 with the goal being a process that will consistently deliver (Active Pharmaceutical Ingredient (API) that meets the Critical Quality Attributes (CQAs).

ST-246 drug product consists of hard gelatin capsules containing 200 mg of active ingredient along with few inactive ingredients. All inactive ingredients are generally accepted as safe (GRAS) and USP/NF excipients. Several small scale drug product batches have been completed for clinical and development purpose.

A series of QbD experiments were completed to understand the drug product manufacturing process. A systematic approach using QbD per ICH guidelines (Pharmaceutical Development Q8(R2), Quality Risk Management Q9) along with the knowledge and experiences gained from the prior lab scale batches was used for the drug product development of ST-246 capsules. Moreover, a continuous quality improvement during the entire product life cycle, including formulation and manufacturing process of ST-246 capsules is intended.

The QbD approach included identification of Critical Quality Attributes (CQAs), critical steps and process parameters in the manufacture of the drug product, and establishment of a design space.

For the construction of a control strategy for the final manufacturing process and quality assurance of ST-246 capsules, the following approaches were employed: Setting the Target Product Profile (TTP), development of an Ishikawa (fishbone) diagram which identifies the potential variables that can have an impact on the desired quality attributes, initial (or early phase) risk assessment of ST-246 drug product manufacturing process, QbD studies using Design of Experiments, assessment and construction of Design space as a control strategy Failure Mode Effect Analysis (FMEA) and Risk Analysis and Evaluation: This includes identification and ranking of process variables based on probability, severity and detectability, and determination of the risk score or risk product number (RPN) score and construction of Pareto chart.

Based on various QbD studies, design space and Critical Process Parameters were identified for drug product manufacturing process. The manufacturing process used for drug product is simple and has been scaled-up to one-tenth commercial scale. Three NDA registration batches at pilot scale were successfully completed and process optimization at full commercial scale was also completed successfully. The pilot scale batches and early clinical lots were staged on stability per ICH guidelines in the final packaging containers. Drug product is found to be stable for more than one year at controlled room temperature demonstrating that ST-246 product is stable and can be stored at room temperature.

## 7. Animal Efficacy

Since smallpox is no longer found in nature, human clinical trials designed to link antiviral efficacy to clinical outcome have been replaced by antiviral efficacy evaluations in animal models of orthopoxvirus disease. The FDA has established guidance “Animal Efficacy Rule” (21 CFR Parts 314 and 601) for developing animal models to link efficacy data to clinical correlates predictive of human disease outcome. A number of animal models of orthopoxvirus disease have been developed to evaluate anti-poxvirus compounds. While these models are useful for evaluating antiviral activity of compounds in animals, each model by itself fails to capture all aspects of human disease and, therefore cannot be predictive of clinical outcome. Thus, multiple models of orthopoxvirus infection will be required to evaluate antiviral efficacy of poxvirus inhibitor compounds.

Perhaps the most relevant animal models of orthopoxvirus infection use host-adapted viruses where replication at the periphery and spread is dependent upon the host response to infection and the ability of the virus to counteract this response. In mice and rabbits infected with ectromelia and rabbitpox virus, respectively, lethal infection can be established with as little as 1 pfu of virus delivered by intranasal administration. However, animals die before development of the rash/lesional disease that is the hallmark of smallpox.

### 7.1. Clinical Features of Smallpox

The clinical course of smallpox is presented to better understand the relevance of pathogenesis in various animal modes to the human disease. The life-cycle of variola virus resembles that of other orthopoxviruses in which infection of the natural human host results in virus replication at the periphery followed by systemic spread. The spread of variola in humans has been inferred from animal studies, especially those conducted in mice infected with ectromelia virus [1]. Variola virus is thought to enter the respiratory tract via aerosolized droplets, seeding mucous membranes and passing rapidly into local lymph nodes. Based on animal studies, virus replicates in the local lymph tissue to produce a primary viremia. Virus then travels to the spleen, liver, and reticulo-endothelial system where replication in these organs produces a secondary viremia which is accompanied by disease onset.

Clinical latency ends with the rapid onset of severe headache, backache, and fever, termed the prodromal phase. The prodromal phase correlates with a secondary viremia in which infectious virus can be detected in the mucous membranes of the mouth and pharynx [51]. In animal models of orthopoxvirus infection, the secondary viremia is measured by quantitative PCR (Q-PCR) to determine the amount of viral DNA in blood and tissue samples [52,53]. The virus invades the capillary epithelium of the dermal layer in skin, perivascular cells, and epidermis where replication results in necrosis and the formation of a rash. The spleen, lymph nodes, liver, bone marrow, kidneys, and other viscera may contain large quantities of virus based upon data from animal studies. Replication in the epidermis may be enhanced by secretion of virus-specific growth factors that bind to cellular receptors on keratinocytes and stimulate growth [54].

Infection results in 30% mortality for variola major and 1% for variola minor with the cause of death attributed to toxemia, associated with immune complexes, and hypotension. Toxemia is a poorly defined clinical condition thought to be caused by an excessive inflammatory immune response similar to septicemia associated with systemic bacterial infections. Examination of dermal layers of blood vessels from autopsy patients infected with variola virus shows extensive leakage of the endothelial layer consistent with the presence of high levels of pro-inflammatory cytokines [55].

### 7.2. Disease Symptoms that Define Orthopoxvirus Infections

The current CDC clinical case definition for smallpox is listed as “An illness with acute onset of fever >101 °F (38.3 °C) followed by a rash characterized by firm, deep seated vesicles or pustules in the same stage of development without other apparent cause.” [56]. Laboratory confirmation is based upon polymerase chain reaction (PCR) assay or culture of variola virus from patient tissue. Thus, lesion formation and detection of viral DNA are early symptoms that are used in differential diagnosis of smallpox disease. Moreover, smallpox disease severity is characterized by the extent and appearance of smallpox lesions [1].

#### 7.2.1. Lesion Formation

Skin lesions are the hallmark of orthopoxvirus infection and formed the basis of clinical diagnosis of smallpox infection [51]. Skin lesions or pocks, are formed by infection of the capillary epithelium of the dermal layer of the skin by circulating virus during the secondary viremia phase of infection. The lesions that develop are similar in appearance in humans and non-human primates (NHP) infected with variola virus or monkeypox virus providing a link between human disease and NHP models of orthopoxvirus infection.

A comparison of the pathophysiology of skin lesion formation from smallpox patients, a monkeypox patient, and NHPs infected with MPX reveals a remarkable similarity in the histological changes associated with lesion formation [55,57,58]. The process of lesion development in all cases begins with the productive infection of endothelial cells in the blood vessels within the papillary dermis. Infection leads to dilatation of the capillaries followed by endothelial swelling of the dermal blood vessel walls. Virus spreads to the overlying epithelium and replication in this tissue initiates the characteristic rash associated with orthopoxvirus infection. This process results in the formation of a papule that is characterized by swollen, degenerating cells in the middle layer of the epidermis. Virus-induced inclusion bodies or Guarnieri bodies can be detected in the cytoplasm of degenerating cells. The nuclei of these cells condense and ultimately disappear due to lysis. The cell membranes rupture giving rise to multiloculated vesicles, which increase in size as more cells become involved. The basal layers of the surrounding vesicles proliferate and may be twice the size of the unaffected epidermis giving rise to the elevated border surrounding the vesicle. This appears as a raised area on the skin surrounded by unaffected skin.

The pustule is formed by infiltrating polymorphonuclear granulocytes that degenerate within the vesicle and their nuclei fragment forming a cavity at the center of the lesion. Umbilication, a hallmark of orthopoxvirus lesions, is thought to be caused by swelling of the cells surrounding the cavity through edema and reticulation and proliferation of the basal layers surrounding the vesicles. The proliferating cells surrounding the lesion encroach upon the cavity to form the raised edges of the lesion with a depression in the center as fluid drains from the cavity. During the healing stage, proliferating cells from the surrounding lesion encroach upon the cavity to form parakeratotic cell layer which upon desiccation of the cavity initiates the encrustation process. The parakeratotic cell layer increases in density as the lesion heals. Finally the scab is shed, revealing newly formed epidermis.

The appearance and extent of lesion formation provides a useful marker of orthopoxvirus disease severity and links human smallpox to orthopoxvirus disease in NHP. Lesion development requires systemic virus spread and productive infection of the capillary epithelia. Antiviral therapies that reduce lesion formation in NHP models of orthopoxvirus disease will likely be effective treatments for human smallpox.

#### 7.2.2. Viremia

Systemic disease is a characteristic of smallpox and is caused, in part, by the host response to generalized virus infection. While the primary site(s) of infection in humans has not been well defined, studies of mousepox, rabbitpox, and monkeypox indicate that virus replication occurs predominantly in the reticuloendothelial system to produce a secondary viremia characterized by high levels of circulating virus in the blood [57,59,60]. In animal models of orthopoxivrus infection, the level of circulating viral DNA in the blood measured by Q-PCR, correlates with disease severity [53,61–64]. While plaque assay can also be used to measure infectious virus, interfering substances found in the blood can confound interpretation of the data making PCR a more reliable assay to measure viremia [65]. In humans, information regarding the level of variola virus DNA in blood from smallpox patients is unavailable since PCR technology did not exist prior to the eradication. However, infectious virus has been cultured from the oral mucosa of smallpox patients during the prodromal phase of infection prior to the onset of lesions suggesting that patients contained high levels of circulating virus [51]. Thus, quantifying viral DNA levels in the blood by Q-PCR is a valid measure of disease severity in animal models of orthopoxvirus infection and provides a link to smallpox in humans.

#### 7.2.3. Mortality

Death is often a primary endpoint in animal models of severe orthopoxvirus disease. The cause of death in these experimental systems is not well understood and has been attributed to severe bronchopneumonia, multi-organ failure, and septic shock syndrome [57,66,67]. In humans, mortality due to smallpox has been attributed to bronchopneumonia and toxemia, a poorly defined clinical syndrome that resembles bacterial septicemia [1,55]. While differences may exist in the primary cause of death between infected humans and animals, mortality as an endpoint is still a useful measure of disease severity in animal models of orthopoxvirus infection in that it reflects excessive viral replication in the host. Moreover, therapies that limit virus replication and spread reduce mortality. Animal models that don’t produce 100% mortality require larger treatment and control populations to show statistical significance of the treatment effect. Thus, mortality in these models is less useful than other endpoints for evaluating therapeutic efficacy of an antiviral product.

### 7.3. Efficacy of ST-246 in Small Animal Models of Orthopoxvirus Disease

Models of orthopoxvirus disease were developed in mice, including BALB/c, NMRI, ANC/R and Nu/nu, rabbits, prairie dogs, and ground squirrels (reviewed in [68]). These models provided opportunities to evaluate the antiviral activity of ST-246 against multiple species of orthopoxviruses, including vaccinia virus strains IHD-J, Lister, and WR, ectromelia virus, strain Moscow, cowpox virus, rabbitpox virus, and monkeypox virus [17,69–71]. Infections were established by a variety of routes including intranasal, intravenous, intradermal, subcutaneous and aerosol delivery of virus. In all cases, ST-246 protected animals from severe disease and death. ST-246 treatment has been demonstrated to inhibit poxvirus dissemination virus shedding and systemic disease in mice [72]. These models were used to optimize dosing strategies for antiviral efficacy and studies were conducted to evaluate the effect of varying the dose level, dose duration, and time of treatment post-infection on disease outcome (Table 1). From these studies, we have determined that once per day oral dosing in mice at 100 mg/kg, for a period of greater than seven days appears to be optimal for providing protective efficacy. Treatment can be initiated as late as 72 hours post-infection for full protection. In one experiment in prairie dogs infected with monkeypox virus, treatment initiated 10 days post-infection resulted in 100% protection from death. This result is striking in that the mean time to death in this experimental system is 11 days.

Mice that survive lethal infection due to ST-246-treatment are resistant to subsequent challenge with lethal doses of vaccinia virus due to acquisition of protective immunity during the initial infection [17]. ST-246 has also been shown to protect in mice from lethal infection that are deficient in either CD4+ or CD8+ T cells, but not both, regardless of the presence or absence of B-cell deficiency [73]. ST-246 treatment in combination with smallpox vaccination does not appear to diminish the immune response raising the possibility that ST-246 could be co-administered with the smallpox vaccine to reduce vaccine-related side-effects and protect individuals from infection prior to acquisition of protective immunity [74]. Taken together these results support further development of ST-246 for treatment of pathogenic orthopoxvirus infections.

### 7.4. Non-human Primate Models of Orthopoxvirus Infection

**Figure 4.**
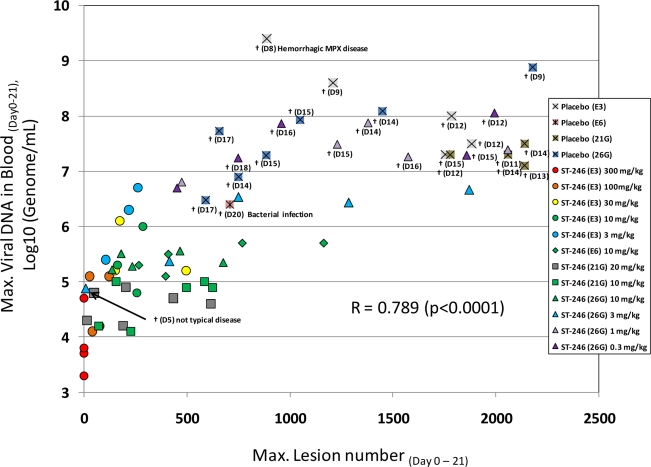
Correlation between maximum monkeypox viral DNA levels in the blood *versus* maximum lesion number in non-human primates infected with monkeypox virus. Viral DNA levels were determined by quantitative PCR assay. Maximum viral DNA levels and lesion number were determined over a period of 21 days starting at the time of infection (Day 0). All doses were administered by oral gavage. Dose levels ranged from 0.3 mg/kg/day to 300 mg/kg/day for 14 days. Treatment was initiated on Day 1 (E3), Day 5 (E6), Days 3 or 4 (21G) or lesion onset on Day 3 or 4 (26G). Studies 21G and 26G were randomized, double blind, placebo-controlled studies conducted in compliance with GLP guidelines. Pearson’s correlation coefficient was calculated to show the degree of correlation.

Primate models of orthopoxvirus disease have been developed to evaluate efficacy of new smallpox vaccines and antiviral drugs. Inoculation of cynomolgus macaques with monkeypox virus or variola virus delivered by intravenous or intratracheal injection produces a lethal (or semi-lethal in the case of variola virus) infection that reproduces the lesional disease characteristic of smallpox and monkeypox virus infection in humans [53,63,75]. In fatal cases of monkeypox or variola virus infection, animals die between 10 and 18 days post-infection with over 750 poxvirus lesions. The extent of the pox rash correlates with disease severity in humans based on the World Health Organization (WHO) grading scale of mild disease, 5–25 lesions; moderate disease, 26–100 lesions; severe disease, 101–250 lesions; and grave disease, >250 lesions. This model appears to mimic disease produced during the secondary viremia phase of human smallpox. In addition, there is a direct correlation between lesion number and viral DNA in the blood (Figure 4) and the level of viral DNA is correlates well (p < 0.05) with the mean time to death in the IV monkeypox model (data not shown).

**Figure 5.**
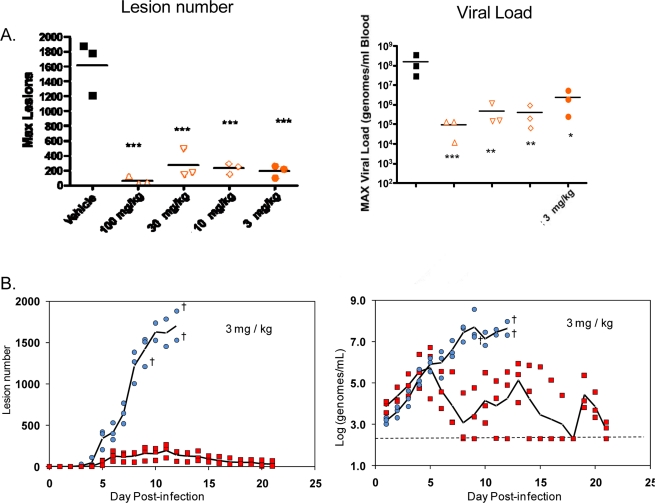
Efficacy of ST-246 in a non-human primate model of monkeypox virus infection. Cynomolgus macaques were infected with 5 × 10^7^ pfu of monkeypox virus via intravenous inoculation. At 3 days post-inoculation, ST-246 was administered by oral gavage once per day for 14 consecutive days. Viral DNA in the blood was determined by quantitative PCR. Viral DNA and lesion counts were measured every day for 21 days (adapted from Jordan *et al.*) [63]. **(A)** The maximum viral DNA levels in the blood and lesion number for individual animals over the course of the experiment were plotted per treatment group. The average value (bar) for each treatment group is shown. **(B)** Daily lesion numbers and viral DNA levels in the blood were shown for vehicle-treated animals (blue circles) and ST-246-treated animals at 3 mg /k g (red squares) with the average values plotted as a single black line. Cross symbols indicate day of death. The dashed horizontal line shows the limit of quantification of viral DNA.

Infection of non-human primates (NHP) via intravenous injection (IV) of monkeypox virus has been used to evaluate efficacy of ST-246 [63,64]. ST-246 administered at three days post-infection (dpi) at four different doses, from 100 mg/kg down to 3 mg/kg, once a day for 14 days, protected NHP 100% from a lethal infection with monkeypox virus (MPX) and reduced the viral load and lesion formation [64] (Figure 5). In NHP, a ST-246 dose of 10 mg/kg/day for 14 days resulted in blood exposure comparable to levels attained in humans administered 400 mg in the fed state. A randomized double blind, placebo controlled study was conducted to evaluate the efficacy of ST-246 in cynomolgous macaques inoculated with a lethal dose of monkeypox virus via intravenous injection. Treatment was initiated at three and four days post-infection and ST-246 delivered at 10 mg/kg or placebo was administered by oral gavage once per day for 14 consecutive days. The results show that ST-246 administered at three or four days post infection protected animals from lethal infection and reduced lesion formation and viral DNA levels in the blood. In this model, five of the 16 NHPs showed lesion onset on Day 3 while the remaining 11 animals in the study all had lesions by Day 4 post-inoculation [76].

A compilation of the efficacy data from NHP studies conducted with ST-246 shows a strong correlation between viral DNA levels in the blood and lesion formation (Figure 4). Moreover, in all studies ST-246 protected NHPs from lethal infection with monkeypox virus and reduced lesion formation and viral DNA levels in blood even when compound was administered as late as five days post-inoculation. This time point is approximately 24 to 48 hours after initial lesion formation. These results demonstrate that ST-246 provides therapeutic efficacy against lethal monkeypox virus infection of NHP.

ST-246 was also shown to be effective at reducing disease in NHP infected with variola virus [63]. Based upon this preliminary data, a second randomized double blind placebo controlled study was conducted to evaluate the efficacy of ST-246 in cynomolgous macaques infected with variola virus via intravenous injection. In this study treatment, was initiated at lesion onset (between day 3 and day 4 post-infection). The results show that ST-246 reduced lesion numbers compared to placebo-treated animals [77]. Moreover the rate of increase in viral DNA levels in the blood were significantly different between ST-246-treated and placebo-treated animals. While the amount of virus delivered was expected to result in some mortality, all of the animals survived infection. Taken together these results suggest that ST-246 administered at lesion onset at 10 mg/kg can protect monkeys from lethal infection with monkeypox virus and reduce variola virus induced disease.

## 8. Clinical

ST-246 has been shown to be safe and well tolerated in single and multiple dose human clinical trials with exposure levels consistent with once per day dosing [78,79]. The human dose selection process for phase I clinical trials was based on the NOAEL for the monkey converted to the human equivalent dose using the common conversion factors as recommended in FDA guidance 81 and calculated based on body surface area, not direct mg/kg weight. The monkey was selected as the most relevant species based on the single dose NOAEL of 2000 mg/kg, the multiple-dose NOEL of 300 mg/kg/day, and its physiologic similarities to humans. The clinical volunteer starting single dose of 500 mg in the first Phase 1 study was conservatively calculated to be within a 10 fold safety margin. Single and multiple ascending dose studies in healthy human volunteers established that ST-246 was safe and well tolerated with plasma drug exposures in the range predicted to be sufficient for inhibiting orthopoxvirus replication. No severe adverse events (SAEs) were observed, and no subject was withdrawn due to ST-246. The most commonly reported drug-related AE was neutropenia that was found, upon further analysis, not to be treatment related. ST-246 was readily absorbed following oral administration with mean times to maximum concentration from 3 h to 4 h. Absorption was greater in non-fasting volunteers compared to fasting volunteers.

**Figure 6.**
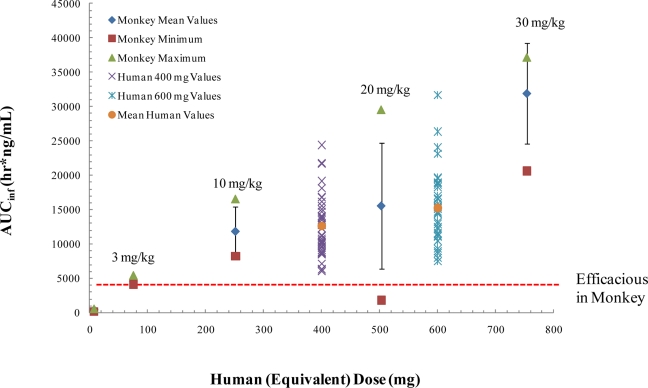
A comparison of ST-246 exposure (area under the concentration time curve, AUC) in monkeys and humans. Monkey doses were converted to human equivalent doses by multiplying first by 0.32 in accordance with FDA Guidance for Industry (entitled Estimating the Maximum Safe Starting Dose in Initial Clinical Trials for Therapeutics in Adult Healthy Volunteers, published July 2005) and then multiplying by the average weight of human (78.6 kg) from the clinical study. The maximum, minimum and mean AUC for monkeys is shown in the green triangles, brown squares, and blue diamonds, respectively. The range in AUC values in humans is indicated by the symbols. The red dashed line indicates the dose of ST-246 that protects non-human primates from lethal infection.

To establish the effective human dose, the exposure levels in humans will have to be comparable to exposure levels in non-human primates that protect animals from lethal orthopoxvirus infection. Based upon data from our preliminary animal efficacy studies using survival as a primary endpoint, an oral dose of approximately 3 mg/kg in non-fasted non-human primates confers 100% protection from death following intravenous injection of 5 × 10^7^ plaque forming units of monkeypox virus strain Zaire [63]. Pharmacological assessment in non-human primates, found that a dose of 10 mg/kg results in blood exposure levels comparable to levels attained in humans administered a 400 mg dose in the fed state (Figure 6). This dose level is well below the no observed adverse effect level (NOAEL) of 2000 mg/kg in a single dose experiment in non-human primates and the no observable effect level (NOEL) 300 mg/kg in a 28-day repeat dose experiment in non-human primates. Given the variability in exposure levels in monkeys and humans in the fed and fasted states, we predict that human doses of 400 mg in the fed state will encompass plasma drug exposure levels comparable to those that provide protective efficacy in the non-human primate model of orthopoxvirus disease.

ST-246 was used to as part of the treatment regimen for several clinical cases of orthopoxvirus infection. ST 246 was used for the treatment of a child with eczema vaccinatum [9]. The patient, a 28 month old male child with a history of eczema and failure to thrive, was exposed to virus through direct contact with a vaccinee. He presented to the emergency room with high fever and severe eczema. Vesicular skin scrapings and viral culture supernatant from vesicles on the child’s skin were obtained and determined to be positive non-variola orthopox virus by polymerase chain reaction (PCR). The child’s condition continued to worsen despite initial treatment with vaccinia immune globulin intravenous (VIG IV) and he exhibited progressive metabolic then respiratory acidosis, hypoalbuminemia, hypothermia, and hypotension. ST-246 was orally administered via a nasogastric tube. The subject also received one dose of Cidofivir and repeated doses of VIG IV. Clinical signs of the child’s improvement were observed within 1 week of the anti-viral intervention (ST 246, Cidofivir, and VI–IV). The patient’s clinical symptoms continued to improve and the child is thriving and continues to do well.

A second case was reported in a 20-year-old male who received the smallpox vaccine and was subsequently diagnosed with acute myeloid leukemia approximately 12 days after vaccination [80]. Chemotherapy was initiated to treat the leukemia caused a severe impairment of immune function resulting in progressive vaccinia six to seven weeks after vaccination. He received topical imiquimod (5%) at the vaccination site and VIGIV was administered intermittently throughout the course of treatment. ST-246 was administered at 400 mg once per day for 15 days and increased to 800 mg for five days and then 1200 mg for approximately two months. CMX-001, an oral prodrug of CDV, was administered at 200 mg approximately 3.5 weeks after diagnosis or progressive vaccinia and 100 mg every week for five weeks. An increase in lymphocyte count correlated with improvement of symptoms and the patient was declared virus free and treatment was discontinued nine weeks after diagnosis.

## 9. Summary

Development of a self-administered orthopoxvirus-specific antiviral drug with superior safety profile and improved pharmacological properties will serve as both a deterrent against a possible biological attack, provide immediate protection to unvaccinated persons in the event of an attack and protect individuals from emerging orthopoxvirus infections. A self-administered antiviral drug would supplement, rather than replace vaccination, since data from a variety of animal experiments indicate that systemic therapy does not interfere significantly with a vaccination reaction or prevent the development of adaptive immune responses to vaccine antigens. Drug treatment would be especially important for individuals who were vaccinated too late in the incubation period and have not yet acquired protective immunity. Moreover, antiviral therapy in combination with a live virus vaccine could be used to prevent or treat complications arising during the course of a mass vaccination campaign.

Antiviral drugs would be of critical importance as the only effective means of protecting severely immunocompromised individuals exposed to smallpox. Such persons could safely receive highly attenuated vaccines such as MVA, but recent studies in nonhuman primates indicate that they will fail to develop a protective immune response. Antiviral therapies would therefore be the only means of preventing severe or fatal infection. A self-administered drug with broad anti-pox viral activity could also be used to increase the safety of vaccination for the 9–30% of the population with atopic dermatitis/eczema who may be at risk for disseminated vaccinia virus infection (eczema vaccinatum) following administration of the current live-virus vaccine.

## Figures and Tables

**Table 1.** Summary of ST-246 efficacy in animal models of orthopoxvirus disease.

**Virus**	**Host**	**Optimal oral dose mg/kg, s.i.d.**	**Optimal dose duration (% efficacy)**	**Time of treatment (% efficacy)**	**Reference**
Vaccinia	Mouse	100	>5 days (100%]	TBD	[17,69]
Cowpox	Mouse	100	>7 days (93%]	48 h.p.i. (93%)	[69]
Ectromelia	Mouse	100	>5 days (100%)	72 h.p.i. (100%)	[69]
Monkeypox	Squirrel	100	TBD	72 h.p.i. (100%)	[71]
Rabbitpox	Rabbit	40	TBD	48 h.p.i. (100%)	[70]
Monkeypox	Monkey	3	TBD	96h.p.i. (100%)	[64,63]
Variola	Monkey	10	TBD	24 h.p.i. (100%)	[63]

TBD—To be determined.

s.i.d.—Once a day.
